# The complete chloroplast genome sequence of *Stephanandra incisa*

**DOI:** 10.1080/23802359.2020.1867012

**Published:** 2021-02-08

**Authors:** Cui-Ping Zhang, Zheng-Zhao Xu, Xiao-Man Xie, Yi-Zeng Lu, Wei Li, Kui-Ling Wang, Xin-Qiang Jiang

**Affiliations:** a College of Landscape Architecture and Forestry, Qingdao Agricultural University, Qingdao, China; b Shandong Provincial Center of Forest Tree Germplasm Resources, Jinan, China

**Keywords:** *Stephanandra incisa*, chloroplast genome, phylogenetic relationships

## Abstract

*Stephanandra incisa* is a typical discontinuous distribution species in the eastern part of the subspecies with a high economic and ecological value. In this study, we have obtained the complete chloroplast genome of *S. incisa* using high-throughput sequencing. The chloroplast genome length was 159,583 bp, the AT content was 63.7%, while the large single copy and a small single copy area were 88,018 bp and 18,817 bp, respectively. It contains 131 genes, including 86 protein-coding genes, 37 transfer RNA genes, and eight ribosomal RNA genes. A maximum-likelihood phylogenetic tree supported the fact that *S. incisa* is closely related to *Pyracantha fortuneana* and *Amelanchier sinica*, which is consistent with the taxonomic view.

*Stephanandra incisa* (Thunb). Zabel is a deciduous shrub of the Rosaceae subfamily, as a discontinuous distribution species, which mostly found in Eastern China, but rarely in Korea and Japan. It grows from 500 to 1000 m above sea level on the sunny side of slopes (Li 2007). *S. incisa* is often used as medicinal, ornamental, and nectar plant materials. At present, the population regeneration of *S. incisa* is affected by the factors such as disorderly utilization and distribution development (Li et al. 2016). Hence, there is an urgent need to understand the genetic variation of *S. incisa* on developing necessary protection strategies. In this study, the chloroplast genome of *S. incisa* has been fully assembled.

Fresh young leaves of *S. incisa* were collected from the Mountain Lao reserve in Qingdao of Shandong province, China (36°10′N, 120°37′E). The voucher specimen was deposited in Herbarium of the Qingdao Agricultural University (accession number: 20180510SJ03). The total genomic DNA of *S. incisa* was extracted by CTAB methods (Li et al. 2013). The chloroplast genome of *S. incisa* was analyzed by Illumina Novaseq platform. The original data obtained from the Illumina Hiseq sequencing were transformed into sequence data by Base Callingand; and the results were stored in the FASTQ file format.

Approximately, 2.92 Gb of raw data was obtained with paired-end 150 bp read length. The high-quality data were obtained after filtering and removing the low-quality reads. Total of 2.59 Gb clean data was assembled into contigs using SPAdes v3.10.1 (Bankevich et al. 2012). The whole chloroplast genome sequence was annotated with Plastid Genome Annotator (PGA) software (Qu et al. 2019). The sequence of *S. incisa* was submitted to the National Center for Biotechnology Information (NCBI) with the accession number MT683856.

*S. incisa* complete chloroplast genome was divided into four parts including two inverse repeat sequences IRa and IRb (26,374 bp), large single copy (LSC) region (88,018 bp) and small single copy (SSC) region (18,817 bp). The entire GC content was 36.3%. The whole chloroplast genome is a circular DNA molecule with a length of 159,583 bp and 131 genes, which contains 86 protein-coding genes, eight rRNA genes, and 37 tRNA genes.

In order to study the position of *S. incisa* in phylogeny, the plastome of *Hamamelis mollis* (NC 037881) was taken as out-group, and nine published chloroplast genomes of the related species were applied for the phylogenetic analysis using RAxML8.0 (Stamatakis et al. 2008) with maximum-likelihood (ML) method (Figure 1). The ML analysis revealed that *S. incisa* was closely related to *Pyracantha fortuneana* and *Amelanchier sinica.* The chloroplast genome of *S. incisa* would provide useful genetic information for further research on genetic diversity and the conservation of Rosaceae species.

**Figure 1.**
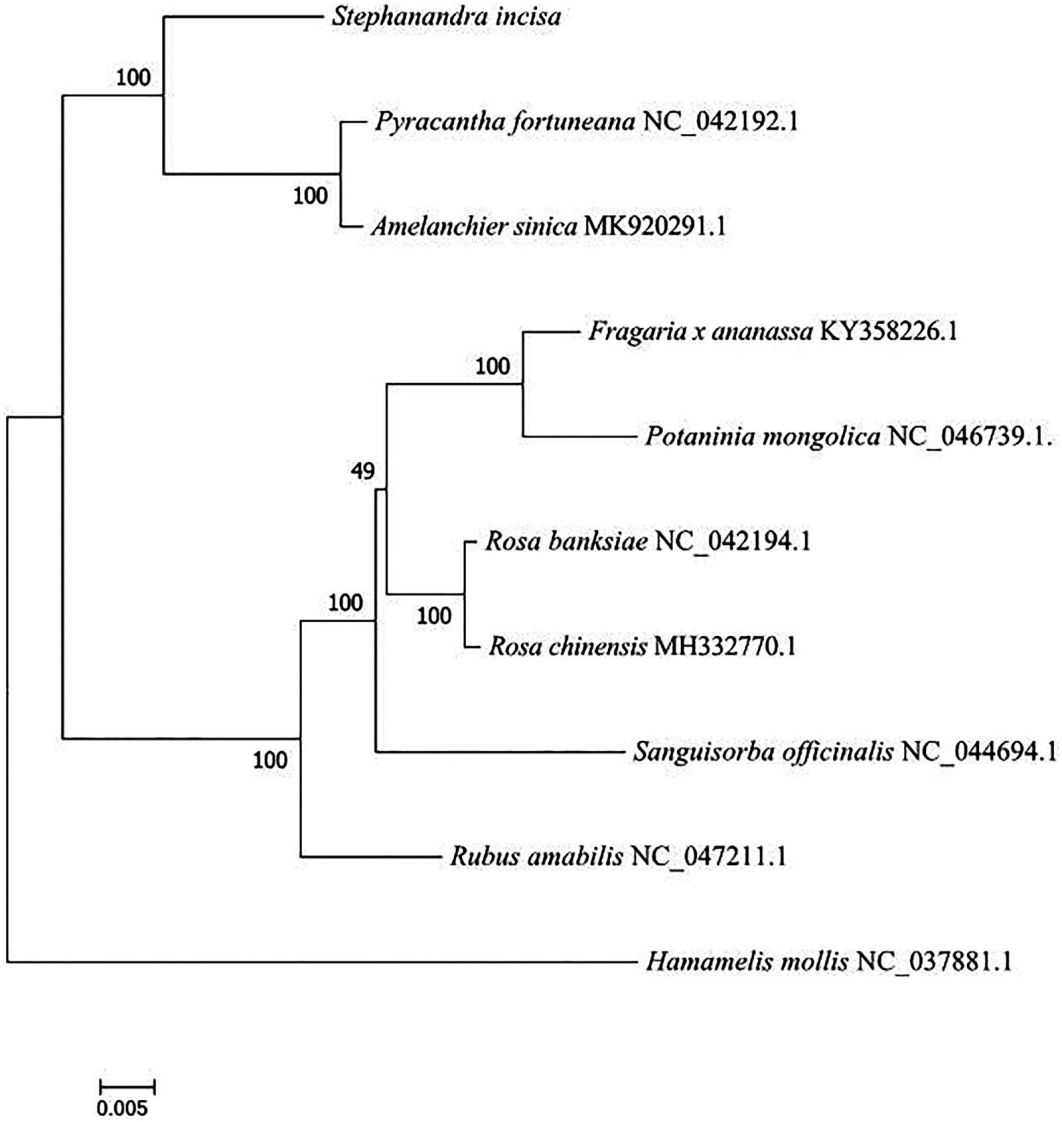
Phylogenetic tree reconstruction of 10 samples using maximum likelihood based on complete chloroplast genome. Accession number: *Pyracantha fortuneana* NC 042192; *Amelanchier sinica* MK 920291; *Fragaria x ananassa* KY 358226; *Potaninia mongolica* NC 046739; *Rosa banksiae* NC 042194; *Rosa chinensis* MH 332770; *Sanguisorba officinalis* NC 044694; *Rubus amabilis* NC 047211; *Hamamelis mollis* NC 037881.

## Data Availability

The data that support the findings of this study are available in GenBank of NCBI at https://www.ncbi.nlm.nih.gov/genbank/, GenBank accession number MT683856. SRA accession number PRJNA678476.
